# Effects of *Trichophyton mentagrophytes* infection on the immune response of rabbits

**DOI:** 10.7717/peerj.7632

**Published:** 2019-09-20

**Authors:** Chenwen Xiao, Guolian Bao, Qiang Wei, Yan Liu, Jiaoyu Wang, Quanan Ji, Yee Huang

**Affiliations:** 1 Institute of Animal Husbandry and Veterinary Science, Zhejiang Academy of Agricultural Sciences, Hangzhou, China; 2 Institute of Plant Protection and Microbiology, Zhejiang Academy of Agricultural Sciences, Hangzhou, Zhejiang Province, China

**Keywords:** Trichophyton mentagrophytes, Immune response indices, Dermatophytosis, Rabbits

## Abstract

**Background:**

Rabbit breeding has developed into a large-scale industry, and as such, the incidence of dermatophytosis in rabbits has become increasingly common. A rabbit model with *Trichophyton mentagrophytes* infection was established to study the changes within the immune responses after fungal infection.

**Methods:**

After the *T. mentagrophytes* challenge on skin, pathogens on the skin were isolated from the rabbits in the fungal infection (FI) groups 20 days. Fungal observation under microscope were carried out. Identification of strains was achieved by polymerase chain reaction (PCR) using the CDR1 gene. The collected anticoagulant blood samples were analyzed for various blood cell parameters. The levels of antibodies, including IgM and IgA, cytokines, including IL-2, IL-6, and macrophage colony-stimulating factor (M-CSF), and soluble CD4 and CD8 in the serum of the FI group vs. the control group were determined independently. RNA isolation from blood samples and fluorescence-based quantitative PCR were carried out for the mRNA level of *M-csf* 20 days after fungal challenge.

**Results:**

Our model resulted in typical symptoms of dermatophytosis on rabbit skin after challenged with fungus. Pathogens isolated from the infected rabbit skin were confirmed to be *T. mentagrophytes* by microscopic examination and PCR. The number of lymphocytes in the blood of the FI group was significantly decreased in comparison to the control group 2 days after the fungal challenge, but was significantly increased in comparison the control group 10 days after the fungal challenge (*P* < 0.01). Platelet counts of the FI group were significantly higher than in the control group at 2 (*P* < 0.05), 10 (*P* < 0.05), and 20 (*P* < 0.01) days after fungal challenge. The red blood cell distribution width of the FI group was significantly increased in comparison to that of the control group at 2, 10, and 20 days after fungal challenge (*P* < 0.01 for all days). The levels of antibodies (immunoglobulin (Ig) M and IgA (*P* < 0.01)), cytokines (interleukin (IL)-6 (*P* < 0.01), macrophage colony-stimulating factor (M-CSF) (*P* < 0.05)), and soluble CD4 (*P* < 0.01) and CD8 (*P* < 0.01) in the serum were significantly different between the FI and control groups. Serum *M-csf* mRNA level of the FI group was significantly higher than the control group 20 days after fungal challenge (*P* < 0.01).

**Conclusions:**

This study demonstrates how the immune system responds to infection with *T. mentagrophytes* and provides potential targets for the prevention and treatment of dermatophytosis.

## Introduction

Rabbit dermatophytosis is caused by filamentous fungi invading the cornified layer of skin and skin appendages. Several pathogens, including *Trichophyton mentagrophytes*, *Epidermophyton floccosum*, *Microsporum gypseum*, *Astragalus propinquus*, and *Candida tropicalis* have been isolated from rabbits with dermatophytosis in clinical practice(Cui et al., 2011). Since rabbit breeding has developed into a large-scale industry, incidence of dermatophytosis in domestic rabbits has become increasingly common, and as such, has become a public health concern.

Studies regarding the incidence of dermatophytosis have mostly focused on pathogenic bacteria (Gao, 2017). There are limited studies investigating the antifungal immune response associated with dermatophytosis (Chang, 2008), including a previous report showing both specific and nonspecific immune responses in cows with fungal infections (Chang, 2008). Avian organisms mainly rely on effector cells with immune function to resist *Aspergillus* infection. Histological studies have found that any *Aspergillus* pulmonary infection has characteristic neutrophil and monocyte invasion (Wang, Lv & Chai, 2007). Most studies focus on the pathogenesis and immunity of deep fungal infections (Cheng et al., 2016). Phagocytes attempt to kill or destroy fungi in target organs. Other effector cells, such as neutrophils and monocytes, are recruited to sites of infection by the action of inflammatory signals, including cytokines, chemokines and complement components (Shmuel & Levitz, 2015). This study established a *T. mentagrophytes*-induced dermatophytosis model in rabbits in order to detect changes in the immune response indices of infected rabbits, thereby providing insights into the pathogenic mechanism of dermatophytosis and a theoretical basis for the prevention and treatment of dermatophytosis. In this study, the experiment was designed to investigate the immune response of rabbits infected with *T. mentagrophytes.*

## Materials & Methods

### Experimental animals

Twenty healthy two-month-old female New Zealand rabbits (1.5 kg–2 kg) were purchased from Zhejiang Academy of Agricultural Science (Zhejiang Province, China) and were randomly divided into two groups: FI group (*n* = 10) and control group (*n* = 10). All animal experiments in this study were approved by the Animal Ethics Committee of the Zhejiang Academy of Agricultural Science (No:1905). All the animals were accommodated in single cage (100 cm*80 cm*80 cm) in natural condition. The feeding water (ad libitum) and food (purchased from Science and Technology Rabbit Farm of Zhejiang Academy of Agricultural Sciences) were clean and safe.

### Fungal challenge on skin

*T. mentagrophytes* was preserved in our laboratory and was verified by the Institute of Dermatology, Chinese Academy of Medical Sciences, and the Peking Union Medical College, (Nanjing, China). *T. mentagrophytes* was cultured on Sabouraud dextrose agar (SDA) (Beijing Solarbio Science & Technology Co., Ltd., Beijing, China) plates for 2 weeks at 28 °C with 60% moisture and then washed with physiological saline and filtered through three layers of filter paper in order to adjust the number of spores to 1 × 10^8^spores/mL.

In order to prepare the rabbits for the fungal skin challenge, the rabbits were anesthetized by a subcutaneous injection of 1% dicaine before fungal challenge within 5 min in laboratory. A 4 × 4 cm^2^ square in the middle of the back of the neck of each rabbit in the FI group was shaved with a razor. The shaved skin was then rubbed with sand paper (200 mesh) until it was red, but not bleeding. The skin was then sprayed with 75% ethanol, which was allowed to evaporate before subsequently applying one mL of *T. mentagrophytes* spores (1 × 10^8^ spores/mL) on the prepared skin. After the end of the experiment all the animals were preserved on one side of the cage and euthanized by intravenous injection of 100 mg/kg of sodium pentobarbital (Sigma-Aldrich cas no: 57-33-0), resulting in painless death of experimental animals.

### Isolation and cultivation of fungi from the challenge site

Pathogens on the skin were isolated from the rabbits in the FI groups 20 days after the fungal challenge by collecting the dander aseptically on SDA plates. The plates were incubated in a 28 °C incubator with 70% humidity for one week, after which the morphology of the colonies was observed.

### Fungal observation under microscope

The colonies grown on the SDA culture plates were observed under a microscope. A small amount of hyphae were picked up with a toothpick and smeared on a glass slide with a drop of distilled water. The fungi were then observed using an Olympus IX81 (Olympus, Tokyo, Japan) at a magnification of 600 ×.

### DNA extraction and PCR of cerebellar degeneration-related protein 1 (*cdr1*) gene

In order to isolate DNA from the fungi growing on plates. Three milliliters of the 10% working solution was added to the fungi and two steel beads and vortexed at 60 Hz for 90 s. A 1-mL suspension of each sample was transferred into a new Eppendorf tube, boiled in a water bath for 10 min, and subsequently centrifuged at 10,000 rpm for 5 min. The supernatant was collected for using as the DNA template in PCR.

The sequence of the *cdr1* gene obtained in our previous study was used for primer design (Detail data of cerebellar degeneration related protein (CDR)1 were deposited in the Sequence Read Archive of the National Center for Biotechnology Information (NCBI) (accession number GSE80604; SRA: SRP073785). It has been previously demonstrated that altered expression cerebellar degeneration related protein (CDR)1 is associated with the resistance mechanisms (Feng et al., 2018). The detailed information about this gene was provided in supplemental file. The specific primer sequences are listed in Table 1. The 2 × Taq PCR Master Mix was purchased from Aidlab Biotechnologies Co., Ltd. (Beijing, China). The PCR reaction included 12.5 µL of 2 × Taq PCR Master Mix, forward primer (final concentration 0.2 µm), reverse primer (final concentration 0.2 µm), DNA template (one µg), and 9.5 µL double-distilled water. The PCR reaction was performed in a PCR machine (Tprofessional Standard Gradient Thermocycler, Biometra, Göttingen, Germany). The PCR reaction conditions were as follows: 1 cycle of 94 °C denaturation for 3 min; 30 cycles of 94 °C denaturation for 30 s, 55 °C annealing for 30 s, and 72 °C elongation for 1.5 min; 1 cycle of 72 °C elongation for 5 min; and 4 °C reaction termination. The PCR products were separated by gel electrophoresis on a 1.5% agarose gel at 110 V for 20 min. The gels were observed under a 260-nm ultraviolet lamp (Bio-Rad System GelDoc XR+; Bio-Rad, Hercules, CA, USA) for the appropriate band sizes.

**Table 1:** Primer sequences and amplification fragment sizes of cerebellar degeneration related protein 1 (*cdr1*) gene, macrophage colony-stimulating factor (*M-csf*) and glyceraldehyde 3-phosphate dehydrogenase (*Gapdh*) genes.

Primer name	Sequence	Amplicon size
CDR1p-2-F	CGAAGCCTAAAACTGATGCTCT	279 bp
CDR1p-2-R	CTTGCCCTGGGACTATCTGC	
M-CSFp-1-F	TGCAGCCACATCATCGGGAAC	141 bp
M-CSFp-1-R	GCCCTTCTTCAAGTAGCACACGG	
GAPDHp-1-F	CAGCCGCTTCTTCTCGTGCAG	235 bp
GAPDHp-1-R	TTCCCGTTCTCAGCCTTGACC	

### Periodic acid-Schiff (PAS) staining of tissue sections of FI group

Rabbit skin was harvested 20 days after fungal challenge by intravenously injecting 30 mg/kg 3% sodium pentobarbital solution before dissection. The skin tissue specimens were fixed in neutral-buffered 30% formalin (pH 7.4) for 12 h. The specimens were then rinsed in tap water for 30 min, dehydrated one time for 30 min in 80% ethanol, three times for 1 h each in 95% ethanol, twice for 1 h each in 100% ethanol, and then cleared twice for 20 min each time in xylene solution. The tissues were then soaked in paraffin for 3 h at 58–60 °C and then paraffin-embedded, serial sectioned at 4-µm thickness, and placed on glass slides. The slides were heated at 60–62 °C in an incubator (GNP-9160 Thermostatic incubator) for 2 h before staining with hematoxylin and eosin and PAS.

PAS staining was performed after the tissues were deparaffinized twice and rehydrated with an alcohol gradient (3 min in 100% ethanol twice, 2 min in 95% ethanol, 2 min in 80% ethanol, and 2 min in 70% ethanol) and then rinsed in water. The tissue sections were then reacted in 0.5% periodate solution at room temperature for 10 min, rinsed in water, washed with distilled water for 5 min, and stained in Schiff reagent at room temperature for 15 min. The slides were then rinsed in tap water, and the nuclei were counterstained with hematoxylin solution. The stained tissue sections were dehydrated for twice for 3 min each in 95% ethanol, twice for 3 min each in 100% ethanol, twice for 5 min each in xylene solution, and then mounted with neutral gum (Qiujing, Shanghai, China). Fungi with positive PAS staining were red in color with blue nuclear staining.

### Blood sampling and serum isolation

Anticoagulant and procoagulant blood samples were collected from rabbit ear veins 2, 10, and 20 days after the fungal challenge (*n* = 5/group). The collected anticoagulant blood samples were analyzed using the pocH-100i hematology analyzer (Sysmex, Kobe, Japan) for various blood cell parameters, including lymphocyte counts (SCC), platelet counts (PLT), and red blood cell distribution width (RDW). The procoagulant blood samples were used for detecting antibodies produced by the rabbits.

### Antibody and cytokine assay

The levels of antibodies, including IgG, IgM and IgA, cytokines, including IL-2, IL-6, and macrophage colony-stimulating factor (M-CSF), and soluble CD4 and CD8 in the serum of the FI group vs. the control group were determined independently (*n* = 5/group). ELISA (MLBIO, Shanghai Enzyme Linked Biotechnology Co., Ltd. Shanghai, China) (Catalogue Number: IgG: ml027974 ;IgM: ml027243 ;IgA: ml027971 ;IL-2:ml0029781-2; IL-6:ml0029785-2; M-CSF: ml027675) were performed in accordance with the instruction. The detailed procedures were listed as follow: Each well of the microplate had either 50 µL of sample or standard (provided in the kit; MLBIO, Shanghai, China). Then 100 µL of horseradish peroxidase-labeled antibodies was added to the wells and incubated at 37 °C for 60 min. The solution was discarded after the incubation, and the microplate was then dried by patting it on an absorbent paper towel. Washing buffer was added to each well, incubated for one minute, and then discarded. The microplate was again dried by patting it on an absorbent paper towel. The microplate was repeatedly washed for five times, and then 50 µL of substrate A solution and 50 µL of substrate B solution were added into each well. The plate was incubated in the dark at 37 °C for 15 min. The reaction was terminated by adding 50 µL of stopping solution. The OD of each well was measured at a 450 nm wavelength 15 min after terminating the reaction.

### RNA isolation from blood samples and fluorescence-based quantitative PCR

A Simgen kit (Simgen, Hangzhou, China) was used to extract total RNA from the anticoagulant blood samples collected 20 days after fungal challenge (*n* = 5/group). The Simgen’s Reverse Transcription Kit and First Strand cDNA Synthesis Kit (Simgen) were used for the fluorescence-based quantitative PCR. PCR primers were designed by using the NCBI/Primer-BLAST online server. The SYBR Green reaction system (Takara, Kyoto, Japan) was used in the fluorescence-based quantitative PCR strictly in accordance with the manufacturer’s instructions. Primers in the quantitative PCR reaction are listed in Table 1. The reagents used in the PCR reaction included 10 µL 2 × Taq PCR master mix, 0.4 µL forward primer (10 µm), 0.4 µL reverse primer (10 µm), and 0.4 µL ROX Reference Dye II. The PCR reaction conditions were as follows: 1 cycle of 95 °C denaturation for 30 s; 40 cycles of 95 °C denaturation for 5 s, 60 °C annealing for 34 s; and 72 °C elongation for 10 s. Glyceraldehyde 3-phosphate dehydrogenase (*Gapdh*) was used as the endogenous housekeeping control gene. The target and housekeeping genes were analyzed in triplicate, and the average threshold cycle (CT) values were analyzed using the comparative CT method. Gene expression was reported as the fold difference relative to the calibrator. All data were given in terms of relative mRNA expression as mean ± SD.

### Statistical analysis

SPSS 19.0 (IBM SPSS Inc., Chicago, IL) software was used for data analysis in this study. All data were presented as mean ± standard deviation. Comparison between groups was performed using least significant method (LSD) in one-way ANOVA. *P* < 0.05 was considered significant.

## Results

### Symptoms and pathogen identifications

**Figure 1:**
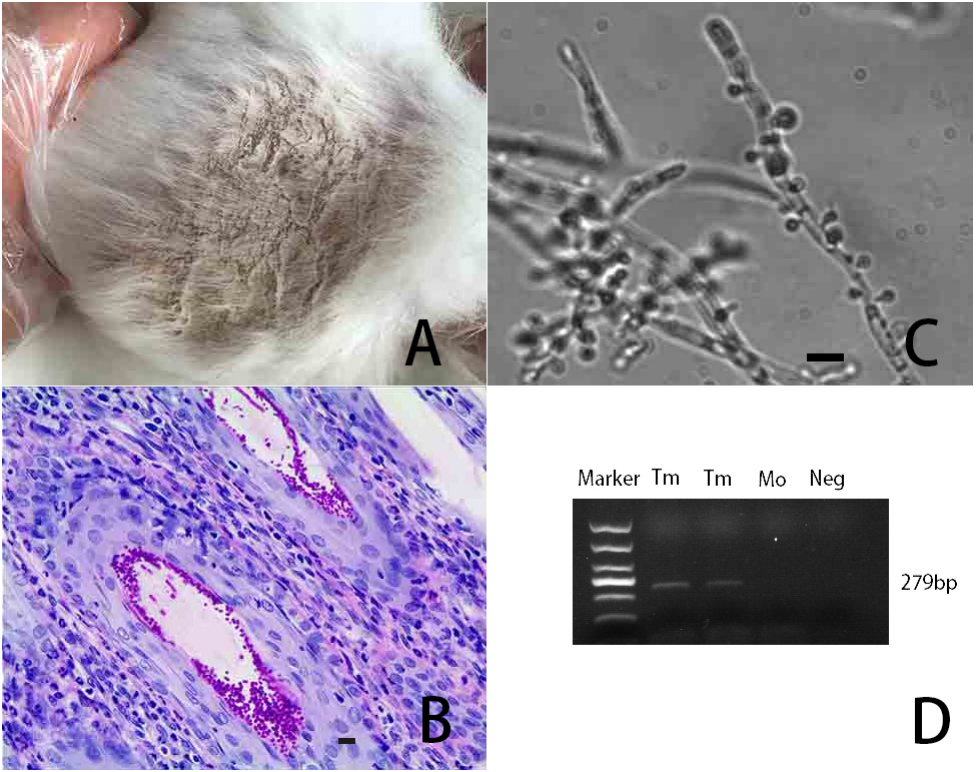
Description of *T. mentagrophytes*. (A) An image showing rabbit skin infected with *T. mentagrophytes* on day 20 after fungal challenge. (B) A representative image of rabbit skin challenged with *T. mentagrophytes* and stained with PAS ** (200×  magnification). Scale bar = 5 µm. (C) The morphology of *T. mentagrophytes* was observed under a microscope (600×  magnification). (D) Gel electrophoresis showing amplification of the fungal cerebellar degeneration related protein 1 (*cdr1*) gene with a size of 279 bp. The lanes marked with Tm represent DNA isolated from *T. mentagrophytes*. The lanes marked with Mo represent DNA from *Magnaporthe oryzae*. The lane marked as Neg represents the DNA-free negative control.

Rabbits in the FI group had severe symptoms of dermatophytosis, including hair loss, grayish-white skin rashes, and reddish skin, 20 days after fungal challenge (Fig. 1A). The health status of animals were not different compared with pre-experiment by naked eyes. As shown in Fig. 1B, PAS staining of the tissue sections indicated a large number of fungi stained red in the hair follicle and extracellular space on the skin with fungal challenge. A large number of hyphae and spores were found under the microscope, and some were stringed and millet-like (Fig. 1C). The hyphae were slender and knotted with small stems on the surface. There were numerous microconidia in single or clumped form. Dander isolated from rabbits in the FI group was positive for *cdr1* DNA, revealing the presence of *T. mentagrophytes* (Fig. 1D).

### Blood cell analysis

**Figure 2:**
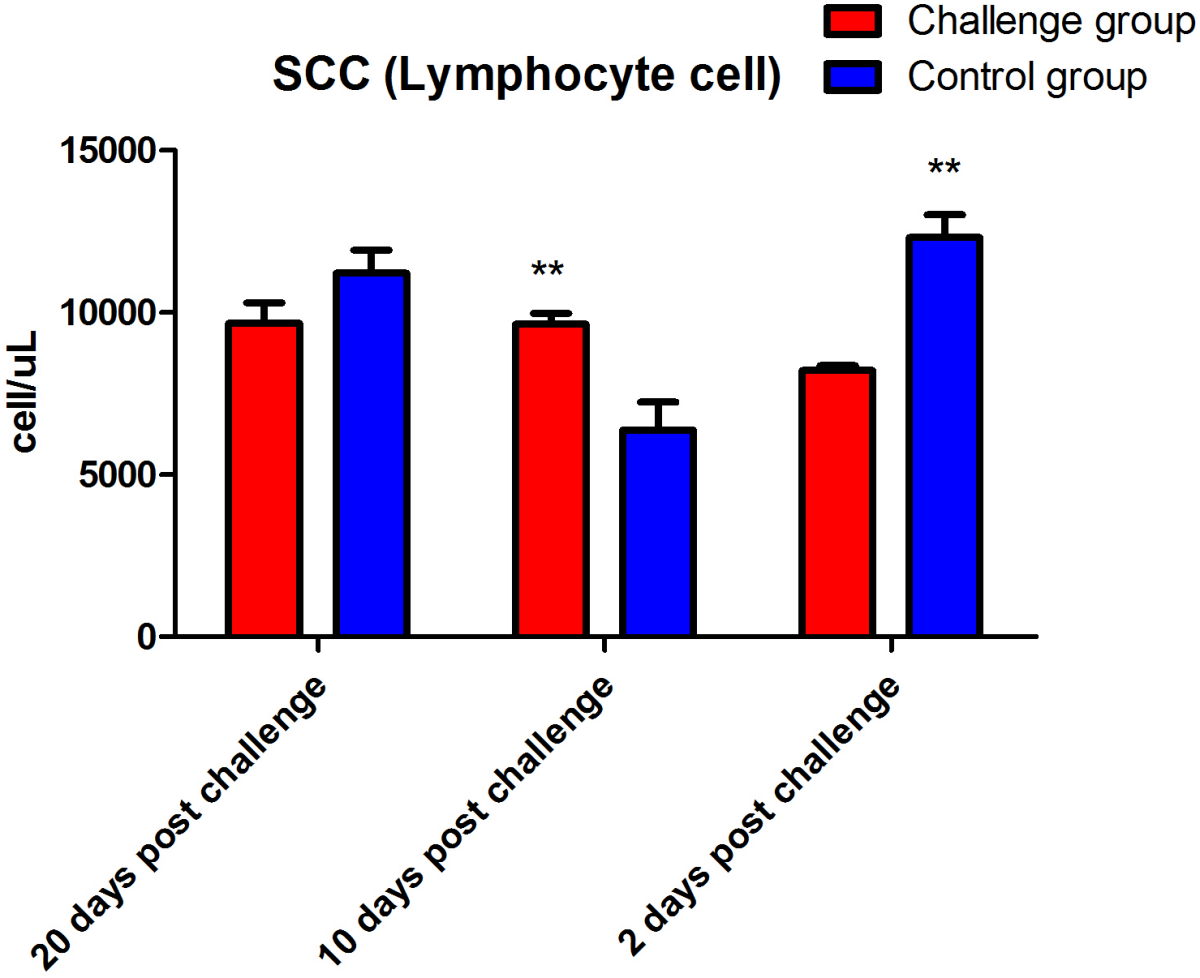
Lymphocyte count after fungal challenge. The lymphocyte count of the fungal infection (FI) group was significantly lower than that of the control group 2 days after fungal challenge (*P* < 0.01), but significantly higher than that of the control group 10 days after fungal challenge (*P* < 0.01). Note: ** represents *P* < 0.01 between the two groups.

**Figure 3:**
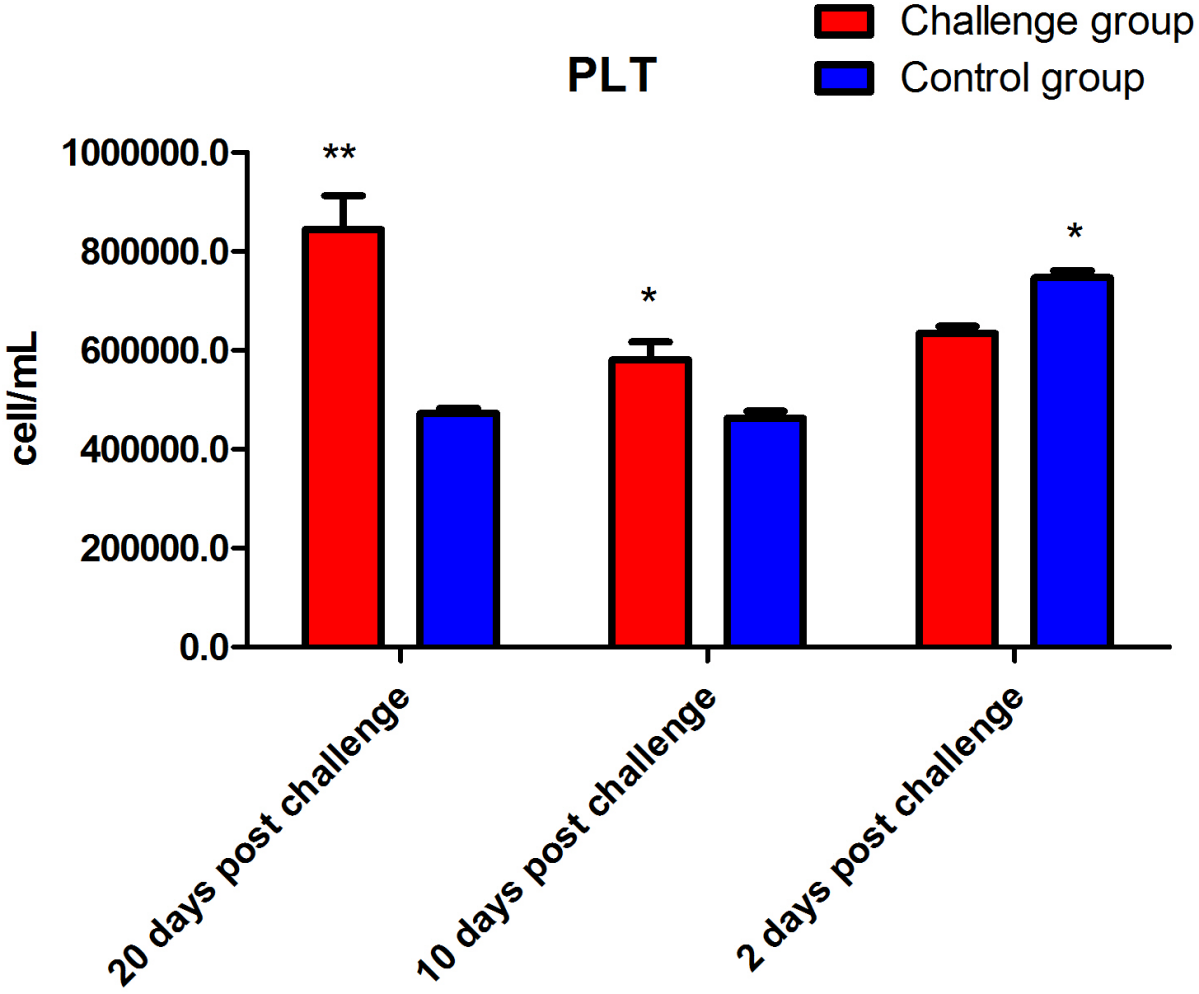
Platelet count (PLT) after fungal challenge. The PLT of the fungal infection (FI) group was significantly lower than that of the control group at 2 days after fungal challenge (*P* < 0.05). In contrast, the PLT of the FI group was significantly increased at 10 (*P* < 0.05) and 20 (*P* < 0.01) days after fungal challenge relative to the control group. Note: * represents *P* < 0.05, and ** represents *P* < 0.01 between the two groups.

**Figure 4:**
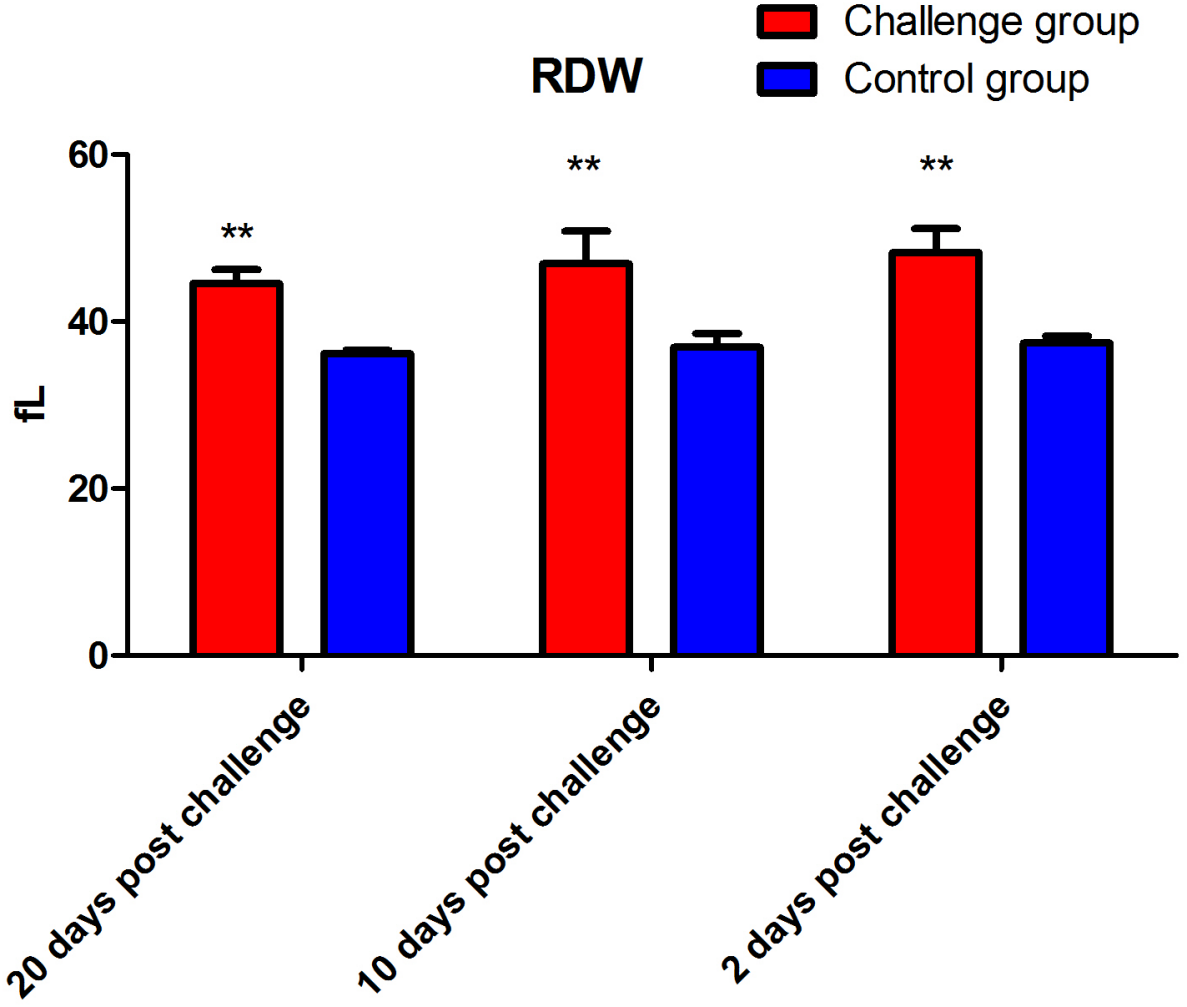
Red blood cell distribution width (RDW) after fungal challenge. The RDW of the fungal infection (FI) group was significantly higher than that of the control group at 2, 10, and 20 days after fungal challenge (*P* < 0.01). Note: ** represents *P* < 0.01 between the two groups.

The data showed that the number of lymphocytes in the FI group was significantly lower than that of the control group (*P* < 0.01) 2 days after fungal challenge. However, the number of lymphocytes in the FI group was significantly higher than that of the control group (*P* < 0.01, Fig. 2) 10 days after fungal challenge. The PLT of the FI group was significantly higher than that of the control group at 2 (*P* < 0.05), 10 (*P* < 0.05), and 20 days (*P* < 0.01) after fungal challenge (Fig. 3). In addition, the RDW of the FI group was significantly higher than the control group from day 2 to day 20 after fungal challenge (*P* < 0.01, Fig. 4). Thus, the data show that *T. mentagrophytes* infection affects the level of lymphocytes, PLT, and RDW in rabbits.

### Antibody and cytokine

**Figure 5:**
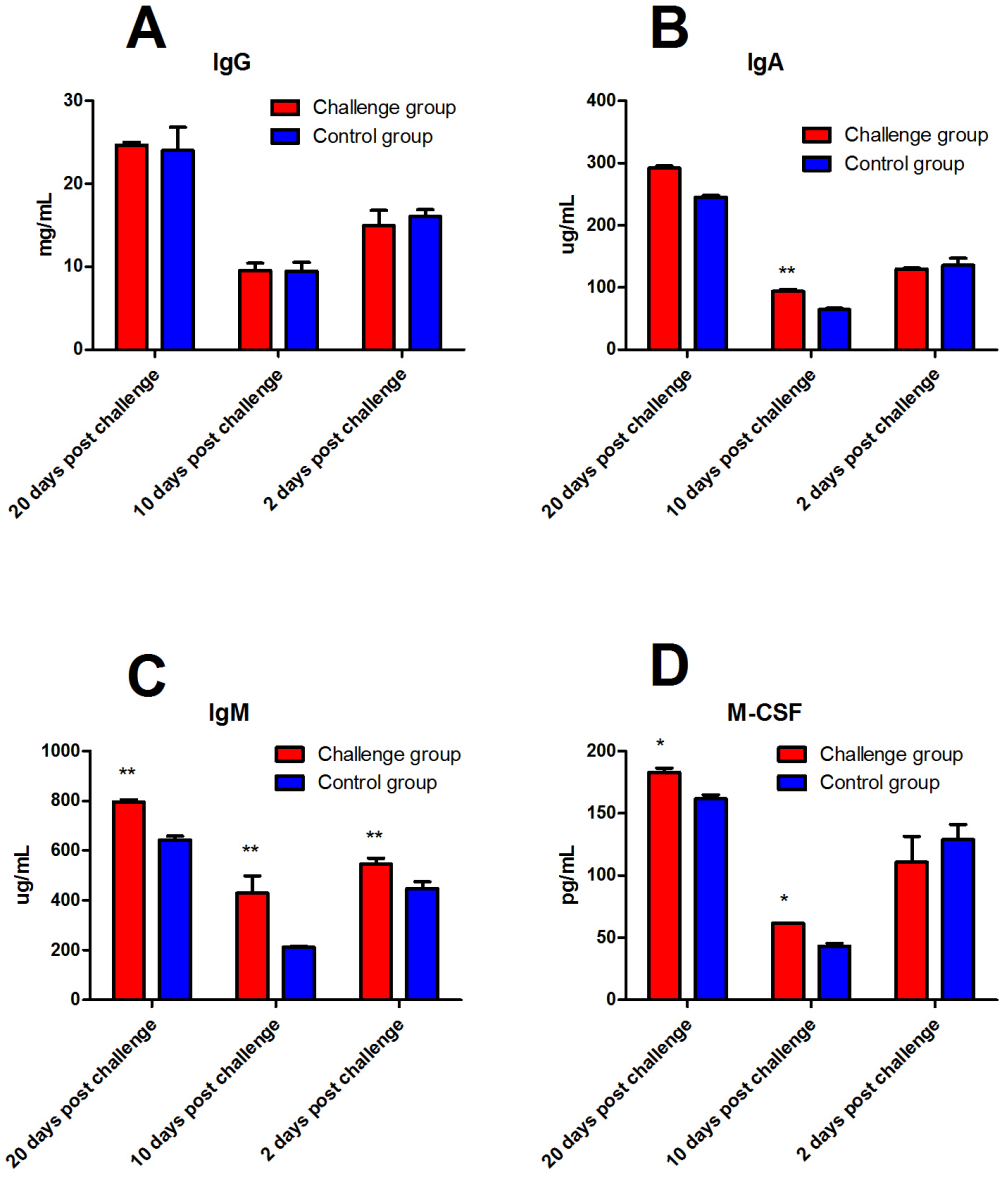
The level of serum antibodies and macrophage colony-stimulating factor (M-CSF) after fungal challenge. There was no significant difference in the serum immunoglobulin (Ig) G levels between the fungal infection (FI) group and the control group. Levels of serum IgA in the FI group were significantly higher than that of the control group 10 days after the challenge (*P* < 0.01). The levels of serum IgM in the FI group were significantly higher than that of the control group at 2, 10, and 20 days after fungal challenge (*P* < 0.01). Serum M-CSF levels in the FI group were significantly higher than that of the control group at 10 and 20 days after fungal challenge (*P* < 0.05). Note: * represents *P* < 0.05, and ** represents *P* < 0.01 between the two groups.

**Figure 6:**
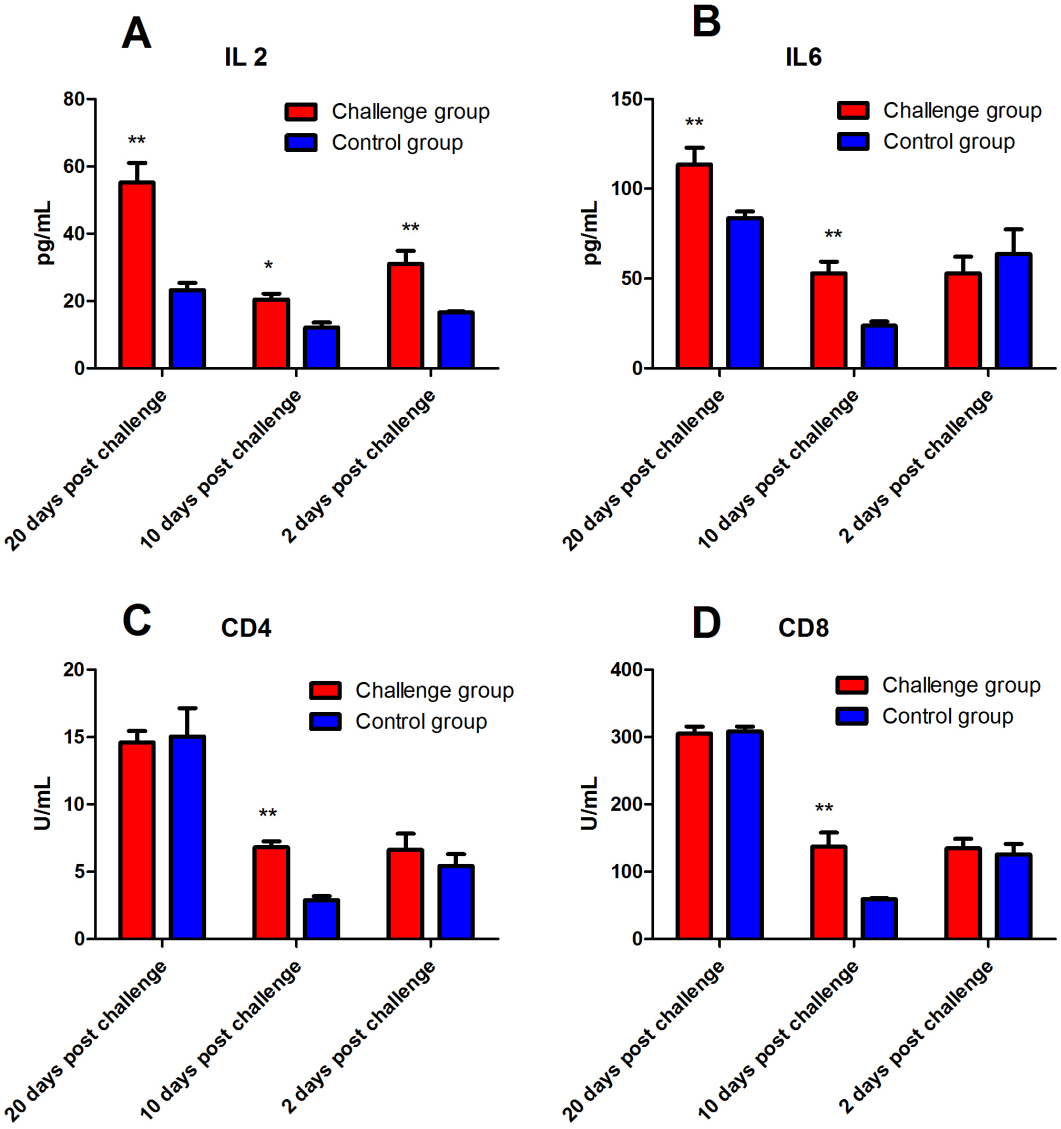
The level of serum cytokines after fungal challenge. Serum interleukin (IL)-2 levels in the fungal infection (FI) group were significantly higher than that of the control group at 2 (*P* < 0.05), 10 (*P* < 0.05), and 20 (*P* < 0.01) days after the fungal challenge. There was no significant difference in serum IL-6 levels 2 days after fungal challenge. However, serum IL-6 levels in the FI group were significantly higher than that of the control group 10 and 20 days after the challenge (*P* < 0.01). In addition, levels of soluble CD4 and CD8 in the serum of the FI group were significantly higher than that of the control group 10 days after the fungal challenge. Note: * represents *P* < 0.05, and ** represents *P* < 0.01 between the two groups.

No significant difference in serum IgG levels was found between the FI and control group. However, serum IgA levels of the FI group were significantly higher than that of the control group 10 days after fungal challenge (*P* < 0.01). In addition, serum IgM levels of the FI group were significantly higher than that of the control groups at 2, 10, and 20 days after fungal challenge (*P* < 0.01, Fig. 5). Serum M-CSF levels of the FI group were significantly higher than that of the control group 10 and 20 days after fungal challenge (*P* < 0.05, Fig. 5). As shown in Fig. 6, serum IL-2 levels of the FI group were significantly higher than that of the control group at 2 (*P* < 0.05), 10 (*P* < 0.05), and 20 days (*P* < 0.01) post-fungal challenge. We did not find a significant difference in serum IL-6 levels between the two groups 2 days after fungal challenge; however, serum IL-6 levels in the FI group were significantly higher than that of the control group (*P* < 0.01) 10 and 20 days post-fungal challenge. Soluble CD4 and CD8 levels in the serum of the FI group were significantly higher than that of the control group 10 days after fungal challenge (*P* < 0.01, Fig. 6).

### Results of RT-PCR

**Figure 7:**
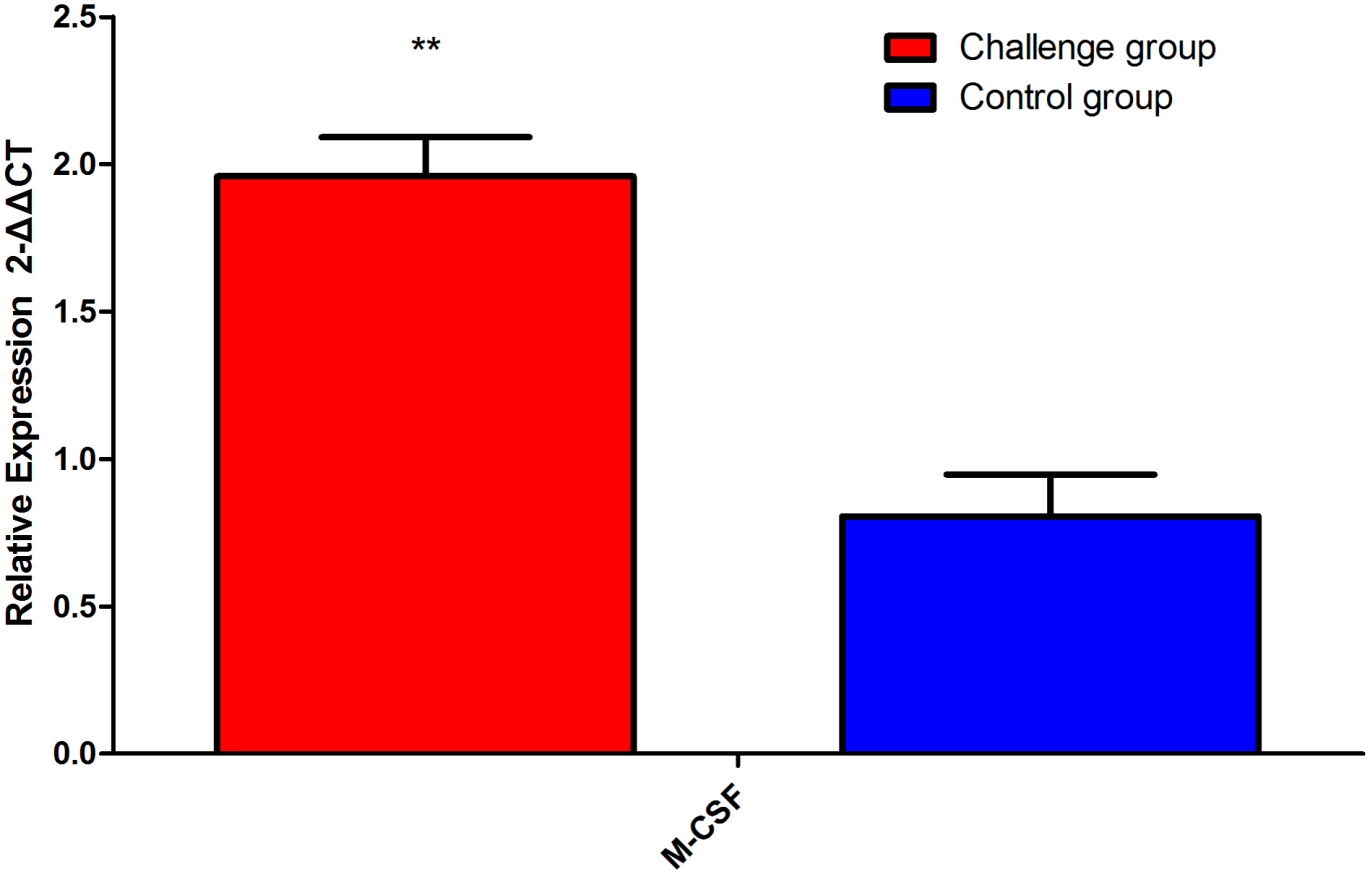
Macrophage colony-stimulating factor (*M-csf*) mRNA level 20 days after fungal challenge. The serum mRNA levels of *M-csf* in the fungal infection (FI) group were significantly higher than that of the control group (*P* < 0.01). Note: ** represents *P* < 0.01.

The mRNA level of *M-csf* in the blood of the FI group was significantly higher than that of the control group (*P* < 0.01, Fig. 7). Thus, our data show that *T. mentagrophytes* infection affects the level of IgA, IgM, and M-CSF in rabbits.

## Discussion

Rabbit dermatomycosis seriously endangers the development of the rabbit industry. However, little research has been performed regarding the effect of fungal infection on the rabbit immune system. In this study, we used transcriptome sequencing results from our previous report to design primer sequences for the *cdr1* gene in order to confirm *T. mentagrophytes* infection in rabbits challenged with *T. mentagrophytes* (Xu et al., 2006). A week after the challenging with *T. mentagrophytes*, the rabbit skin exhibited the typical symptoms of dermatophytosis, including redness, swelling, rough surface, and gray scales in the infected area. The PAS-stained tissue sections demonstrated that the level of *T. mentagrophytes* reached a considerable extent 20 days after fungal challenge, filling the extracellular space and hair follicles within the entire region of the infected skin.

Research regarding immunizations against dermatophytosis provides a valuable basis for the development of prevention and treatment measures. Cell-mediated immune responses play an important role in antifungal immunity. A previous study has shown that cellular immunity plays a role in eliminating fungi in cows with dermatophytosis caused by *Trichophyton verrucosum* (Lepper, 1972). In this study, our results of the blood cell indices did not show a significant difference in the total white blood cell count between the two groups. However, while the lymphocyte count of the FI group was significantly decreased than that of the control group 2 days after fungal challenge, but significantly increased 10 days, which may be because of the variation in the lymphocyte counts of the control group. In this regard, we hypothesize that in the early stage of challenge period, large amount of lymphocytes migrate to the location of the fungal infection, which possibly results in a decrease in the lymphocyte count in blood. However, 10 days after fungal challenge, the lymphocyte count was significantly increased might because the body produced enough lymphocytes to cope with the fungal infection on skin. And the total lymphocytes might be increased dramatically. In addition, this study showed the same changes in the PLT during the skin infection. Platelets are likely to be an important source of inflammatory cytokines (D’Mello et al., 2017; Kullaya et al., 2018). In the presence of platelets, the PBMCs produced less IL-1β, tumor necrosis factor-α, IL-6, and interferon-γ, but more IL-10 (Kullaya et al., 2018). Kullaya et al., 2018 found that platelets had an anti-inflammatory effect on the cytokine response of peripheral blood mononuclear cells (PBMC) incubated with *Mycobacterium tuberculosis*. (Kullaya et al., 2018). In recent years, studies have shown that the clinical value of the RDW is not limited to only the diagnosis of anemia. The RDW is positively correlated with the erythrocyte sedimentation rate and the levels of C-reactive protein, both of which reflect the inflammatory and disease activity within the body (Tecer et al., 2016). In the present study, the RDW of the FI group remained significantly higher than that of the control group after fungal infection present the result of the inflammatory response from the skin infection.

Humoral immunity is one of the important components of the immune response and plays an important role in specific immune functions within the body. Multiple assays including IgG, IgA, IgM, M-CSF, IL-6, CD4 and CD8 show the irregular dynamics at 2 days, 10 days and 20 days after fungal infection, particularly at 10 days , the potential causes might be the immune status of rabbits changed, whether in the attack group or in the control group.

IgG is the most abundant antibody in the serum, accounting for 75–80% of all serum immunoglobulins, and it is also the primary antibody of the immune response (Wu & Liu, 2006). IgG is the main component of humoral immunity in the body and plays a major role in immune protection. After fungal challenge, levels of IgG in the FI group were not different from the control group, which suggests that IgG does not play a major role in the prevention and treatment of fungal diseases. In contrast, the levels of IgA in the FI group were significantly increased 10 days after fungal challenge, suggesting that IgA is involved in the antifungal immune response within the mucosa (Wulandari et al., 2018). IgM is known to play a role in antifungal infection (Lionakis & Levitz, 2018). Tian et al., 2007 reported that IgM is produced against *Candida albicans* infection (Tian et al., 2007). After fungal challenge, the IgM levels in the FI group were significantly increased, suggesting that IgM also plays a key role in humoral immunity against skin fungal infections.

IL-2 mainly stimulates growth and promotes proliferation of T cells. It also promotes the activation, differentiation, and proliferation of natural killer cells (Sun et al., 2002). Our results showed that the IL-2 levels in the rabbits of the FI group were significantly higher than that of the control group 2 days after fungal challenge, indicating that T cells play a role against fungal infection in this animal model. The levels of IL-2 reached the highest on the last day of measurement, indicating that the T cell activity correlated with the severity of dermatomycosis. The source of IL-6 is very broad, and the cells involved in IL-6 production primarily include mononuclear macrophages and activated T cells, specifically Th2 cells (Yu et al., 2016). A previous study also proposed that IL-6 plays a role in antifungal infection (Rose-John, Winthrop & Calabrese, 2017). The increase in the levels of IL-6 may be a result of the cytokines produced by macrophages during the phagocytosis of antigenic materials. The levels of serum IL-6 in the FI group was significantly increased 10 and 20 days after fungal challenge (*P* < 0.01) demonstrated that IL-6 has been shown to modulate the immune response during skin fungal infections. However, in this study the levels of IL-6 in the FI group were not significantly elevated two days after fungal challenge. The specific causes of this slight decrease might be related with the differences of SCC and PLT in the early stage after challenge.

Another factor M-CSF, is one of the biologically active factors produced by macrophages that stimulate hematopoietic progenitor cells to differentiate into monocyte-macrophages and to further maintain the growth, proliferation, and differentiation of the monocyte-macrophages (Guo et al., 2011). Previous study proposed that M-CSF plays a protective role during fungal infections in a neutropenic animal model and plays an important role in antifungal defenses (Kandalla et al., 2016). In present study, serum M-CSF levels of the FI group were significantly higher than that of the control group at 10 and 20 days after fungal challenge, suggesting that M-CSF does play a defensive role during fungal infection. In addition, the mRNA levels of M-CSF in the blood of the FI group was consistent with the M-CSF results in the serum. The biological function of M-CSF is similar to that of IL-6, and both factors are associated with mononuclear macrophages. This further validates the association between M-CSF and IL-6.

CD4 molecules have been considered to play an important role in antifungal infection (Wüthrich et al., 2003), and reduction of CD4 lymphocytes leads to severe and recurrent bacterial and fungal infections (Ghrenassia et al., 2017). A previous study proposed that CD8 may be associated with antifungal infection (Hill & Harmsen, 1991). A recent study has shown that fungal beta-glycan or lipopolysaccharide promoted CD8 T cell activation via autocrine type 1 interferon signaling (Hassanzadeh-Kiabi et al., 2017). Elevation of CD4 and CD8 levels in the FI group after fungal challenge suggests that CD4 and CD8 T cells play a key role in fungal infections on skin tissues.

## Conclusions

We can successfully establish a *T. mentagrophytes* infected model. The lymphocytes, platelet in the blood play an important role after fungal infection in rabbits. The FI model could significantly influence the levels of antibodies [immunoglobulin (Ig) M and IgA (*P* < 0.01)], cytokines [interleukin (IL)-6 ( *P* < 0.01), macrophage colony-stimulating factor (M-CSF) (*P* < 0.05)], and soluble CD4 (*P* < 0.01) and CD8 (*P* < 0.01) in the serum.

##  Supplemental Information

10.7717/peerj.7632/supp-1 Supplemental Information 1 Raw data

10.7717/peerj.7632/supp-2 Supplemental Information 2 RNA test

10.7717/peerj.7632/supp-3 Supplemental Information 3 Blood test raw data

## Additional Information and Declarations

### Data Availability

The following information was supplied regarding data availability: Data is deposited in the Sequence Read Archive: GSE80604, SRA: SRP073785.

## References

[ref-1] Chang H (2008). Effect of the immune function and endocrine of dermatomycosis in cows. Master thesis.

[ref-2] Cheng R, Li D, Shi X, Gao Q, Wei C, Li X, Li Y, Zhou H (2016). Reduced CX3CL1 secretion contributes to the susceptibility of oral leukoplakia-associated fibroblasts to Candida albicans. Frontiers in Cellular & Infection Microbiology.

[ref-3] Cui L, Jiang W, Yang L, Zhang X, Zhang Z, Gao S (2011). Research progress of rabbit fungal dermatopathy. Acta Ecologae Animalis Domastici.

[ref-4] D’Mello C, Almishri W, Liu H, Swain MG (2017). Interactions between platelets and inflammatory monocytes affect sickness behavior in mice with liver inflammation. Gastroenterology.

[ref-5] Feng W, Yang J, Yang L, Li Q, Zhu X, Xi Z, Qiao Z, Cen W (2018). Research of Mrr1, Cap1 and MDR1 in Candida albicans resistant to azole medications. Experimental and Therapeutic Medicine.

[ref-6] Gao Y (2017). The genetic transformation of ZafA gene of Trichophyton mentagrophytes mediated by Agrobacterium tumefaciens. Master’s thesis.

[ref-7] Ghrenassia E, Guihot A, Dong Y, Robinet P, Fontaine T, Lacombe K, Lescot T, Meyohas M-C, Elbim C (2017). First report of CD4 lymphopenia and defective neutrophil functions in a patient with amebiasis associated with CMV reactivation and severe bacterial and fungal infections. Frontiers in Microbiology.

[ref-8] Guo M, Cui W, Huang H, Xu X, Jian H (2011). Granulocyte macrophage-colony stimulating factor and wound healing. Chuang Shang Wai Ke Za Zhi.

[ref-9] Hassanzadeh-Kiabi N, Yáñez A, Dang I, Martins GA, Underhill DM, Goodridge HS (2017). Autocrine type I IFN signaling in dendritic cells stimulated with fungal *β*-glucans or lipopolysaccharide promotes CD8 T cell activation. The Journal of Immunology.

[ref-10] Hill JO, Harmsen AG (1991). Intrapulmonary growth and dissemination of an avirulent strain of Cryptococcus neoformans in mice depleted of CD4+ or CD8+ T cells. Journal of Experimental Medicine.

[ref-11] Kandalla PK, Sarrazin S, Molawi K, Berruyer C, Redelberger D, Favel A, Bordi C, De Bentzmann S, Sieweke MH (2016). M-CSF improves protection against bacterial and fungal infections after hematopoietic stem/progenitor cell transplantation. Journal of Experimental Medicine.

[ref-12] Kullaya V, Van der Ven A, Mpagama S, Mmbaga BT, De Groot P, Kibiki G, De Mast Q (2018). Platelet-monocyte interaction in Mycobacterium tuberculosis infection. Tuberculosis.

[ref-13] Lepper A (1972). Experimental bovine Trichophyton verrueosum infection: preliminary clinical, immunological and histological observations in primarily infected and reinoculated cattle. Research in Veterinary Science.

[ref-14] Lionakis MS, Levitz SM (2018). Host control of fungal infections: lessons from basic studies and human cohorts. Annual Review of Immunology.

[ref-15] Rose-John S, Winthrop K, Calabrese L (2017). The role of IL-6 in host defence against infections: immunobiology and clinical implications. Nature Reviews Rheumatology.

[ref-16] Shmuel S, Levitz SM (2015). The immune response to fungal infections. British Journal of Haematology.

[ref-17] Sun L, Cao X, Zhang M, Zhang W, Liu H (2002). The effect of IL-2 gene modification on the biologic character and function in dendritic cells. Zhongguo Mian Yi Xue Za Zhi.

[ref-18] Tecer D, Sezgin M, Kanık A, Incel NA, Çimen ÖB, Biçer A, Şahin G (2016). Can mean platelet volume and red blood cell distribution width show disease activity in rheumatoid arthritis?. Biomarkers in Medicine.

[ref-19] Tian R, Su W, Zhang X, Liu B, Li W, Liu Y (2007). Detection and analysis of natural antibodies against Candida albicans. Xi Bao Yu Fen Zi Mian Yi Xue Za Zhi.

[ref-20] Wang Y, Lv G, Chai T (2007). Advances in immune responses to avian fungal infections and toxin poisoning. Journal of Dalian University for Nationalities.

[ref-21] Wu X, Liu W (2006). The clinical study of She-dan herbal decoction in treatment of acne vulgaris and its effects on the levels of IgG and IL-2 in peripheral blood of the patients. Zhongguo Pi Fu Xing Bing Xue Za Zhi.

[ref-22] Wulandari EA, Saraswati H, Adawiyah R, Djauzi S, Wahyuningsih R, Lee S, Price P (2018). Evaluation of the protective role for Candida albicans–reactive immunoglobulin a against oral fungal infection. JAIDS Journal of Acquired Immune Deficiency Syndromes.

[ref-23] Wüthrich M, Filutowicz HI, Warner T, Deepe GS, Klein BS (2003). Vaccine immunity to pathogenic fungi overcomes the requirement for CD4 help in exogenous antigen presentation to CD8+ T cells: implications for vaccine development in immune-deficient hosts. Journal of Experimental Medicine.

[ref-24] Xu Z, Zhang LX, Zhang JD, Cao YB, Yu YY, Wang DJ, Ying K, Chen WS, Jiang YY (2006). cDNA microarray analysis of differential gene expression and regulation in clinically drug-resistant isolates of Candida albicans from bone marrow transplanted patients. International Journal of Medical Microbiology.

[ref-25] Yu X, Yang Z, Chu Y, Zhang Y, Yu M (2016). Bioinformatics prediction of IL-6 gene structure and function. International Journal of Laboratory Medicine.

